# PDE4D: A Multipurpose Pharmacological Target

**DOI:** 10.3390/ijms25158052

**Published:** 2024-07-24

**Authors:** Matteo Lusardi, Federica Rapetti, Andrea Spallarossa, Chiara Brullo

**Affiliations:** Department of Pharmacy (DIFAR), University of Genoa, Viale Benedetto XV 3, 16132 Genova, Italy; federica.rapetti@unige.it (F.R.); andrea.spallarossa@unige.it (A.S.)

**Keywords:** phosphodiesterases 4, phosphodiesterases 4D, drug design, neurodegenerative diseases, catechol moiety

## Abstract

Phosphodiesterase 4 (PDE4) enzymes catalyze cyclic adenosine monophosphate (cAMP) hydrolysis and are involved in a variety of physiological processes, including brain function, monocyte and macrophage activation, and neutrophil infiltration. Among different PDE4 isoforms, Phosphodiesterases 4D (PDE4Ds) play a fundamental role in cognitive, learning and memory consolidation processes and cancer development. Selective PDE4D inhibitors (PDE4Dis) could represent an innovative and valid therapeutic strategy for the treatment of various neurodegenerative diseases, such as Alzheimer’s, Parkinson’s, Huntington’s, and Lou Gehrig’s diseases, but also for stroke, traumatic brain and spinal cord injury, mild cognitive impairment, and all demyelinating diseases such as multiple sclerosis. In addition, small molecules able to block PDE4D isoforms have been recently studied for the treatment of specific cancer types, particularly hepatocellular carcinoma and breast cancer. This review overviews the PDE4DIsso far identified and provides useful information, from a medicinal chemistry point of view, for the development of a novel series of compounds with improved pharmacological properties.

## 1. Introduction

Phosphodiesterases (PDEs) are multigene super-family enzymes that hydrolyze the second messenger cAMP and cyclic guanosine monophosphate (cGMP) into AMP and GMP, respectively [1]. Based on their structure, sequence homology, and selectivity for cAMP or cGMP, PDEs are classified into eleven distinct families (namely, PDE1–11) that are further subclassified into many subtypes [1].

The PDE4 isoform was the first discovered variant and preferentially hydrolyses cAMP with low affinity (K_m_ values of 1–6 μM) [2,3]. PDE4 enzymes are abundantly expressed in the brain, immune system cells (e.g., monocytes, macrophages, and neutrophils), and cardiovascular tissue, being involved in a variety of physiological processes, including brain function, monocyte and macrophage activation, neutrophil infiltration, vascular smooth muscle proliferation, and myocardial contractility [4].

PDE4 inhibition elevates intracellular cAMP levels, reducing the expression of inflammatory cytokines such as tumor necrosis factor (TNF), interleukin (IL)-17, and interferon (IFN)-γ and IL-23, and increasing regulatory cytokines, such as IL-10. For the pharmacological potential of its inhibition, the PDE4 isoform is widely investigated in medicinal chemistry [5,6,7,8], particularly as a target for anti-neurodegenerative agents [9].

Four different PDE4 subtypes (namely, PDE4A, PDE4B, PDE4C, and PDE4D) have been identified and further subdivided into about twenty-five different PDE4 variants with specific localization within the cell compartments [10,11]. For instance, the PDE4B subfamily comprises five isoforms (PDE4B1–5), each characterized by different expression levels and functions [12]. Different from PDE4C, isoenzymes A, B, and D are highly expressed in immune system cells [13]. Moreover, PDE4B and PDE4D are involved in neutrophil modulation. The PDE4D isoform is highly expressed in the brain, specifically in the hippocampus, and PDE4D gene-deficient mice exhibit long-term potentiation leading to learning ability and memory increase, as well as nerve regeneration [14]. Consequently, PDE4D appears to be a predominant target for cognitive enhancement, with its inhibition involved in neuroplasticity and neuroinflammation [15].

### 1.1. The Role of PDE in Pathological Conditions

The wide variety of intracellular cyclic nucleotide targets results in an expanding role for PDE isoforms in regulating cellular events [1]. Therefore, the dysfunction of PDE has been associated with several pathophysiological states including those affecting fertility, immunity, cancer, the nervous system, the cardiovascular system, and general metabolism [1,4,9]. The location of a specific PDE isoform directly influences its overall function as well as its catalytic activity. Consequently, the changes in PDE locations based on tissue type, age, or disease status, possibly due to factors such as the activation of receptors; alterations in calcium signaling; or elevations in cyclic nucleotides become of paramount importance when considering the therapeutic potential of a given PDE isoform [9,10]. Furthermore, none of the eleven PDE isoforms share the exact same combination of substrate specificity, tissue expression profile, and subcellular localization. This aspect plays a pivotal role in all the diseases caused by compartment-specific defects in cyclic nucleotide signaling [10]. For instance, the function of soluble guanylyl cyclase seemed to be impaired in Alzheimer’s disease (AD), causing a decrease in cytosolic pools of cGMP, while, in colon cancer, the dysregulation or suppression of membrane-bound guanylyl cyclase and the overexpression of PDE10A led to a reduction in membrane-proximal pools of cGMP [2,4]. Additionally, compartment-specific defects in cyclic nucleotide signaling have been implicated in other pathological states including colorectal cancer, erectile dysfunction, hypertension, cardiac hypertrophy, acrodysostosis, and Huntington’s disease (HD) [1,4,9]. Moreover, a high presence of PDE isoforms in specific brain regions has been demonstrated; for this reason, these enzymes have been widely studied as targets for central nervous system (CNS) disorders [9,10]. As detailed in Table 1, PDE1, PDE2, PDE4, and PDE8 are all expressed in both frontal cortex and parietal cortex, while PDE9 is only present in the frontal cortex. Other brain regions such as the cerebellum (PDE4 and PDE9), temporal cortex (PDE1A, PDE4A, and PDE8B), hippocampus (PDE1, PDE2, PDE4B, PDE4D, PDE8B, and PDE9A), and striatum (PDE1B, PDE2A, PDE4B, and PDE9A) contain various PDE isoforms, while PDE10 is only expressed in the caudate nucleus. Interestingly, PDE4B and PDE4D isoforms are the most delocalized in the CNS, being present also in the thalamus, hypothalamus, and nucleus accumbens [9].

### 1.2. PDE4 Structure

**Figure 1:**
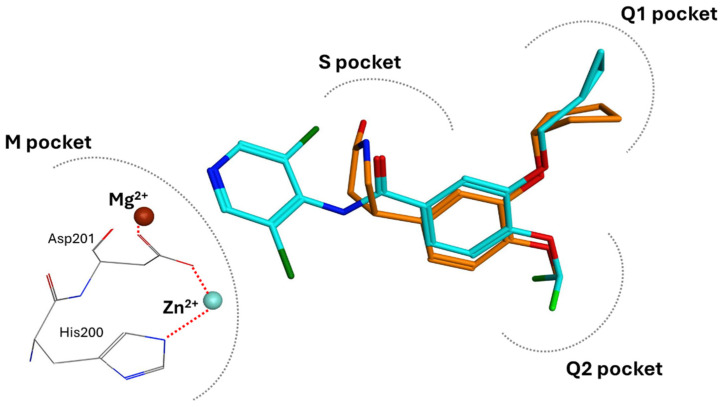
Superposition of Roflumilast (cyan, PDB code: 1XOQ) [18] and Rolipram (orange, PDB code: 1TBB) [23] crystallographic binding modes. The metal M, solvent S, and lipophilic Q1 and Q2 pockets are represented. Details on the coordination of Mg^2+^ and Zn^2+^ ions are reported.

All PDE4 isoforms share a highly conserved catalytic domain (20–45% identity) at the C-terminus, constituted by 300–350 amino acids [16,17]. As assessed by crystallographic studies on PDE4 inhibitors (PDE4Is) [18,19], the catalytic site is sub-divided into three pockets: (i) a metal-binding pocket (M pocket) containing highly conserved hydrophobic and polar amino acids (His and Asp) essential to coordinate Zn^2+^ and Mg^2+^ ions (in detail, two Zn^2+^-binding motifs and one Mg^2+^-binding motif) and necessary to hydrolyze the phosphate moiety of cAMP; (ii) a solvent-filled side pocket (S pocket) characterized by polar residues and filled with water molecules important for the interaction between enzymes and inhibitors; and (iii) two Q pockets (namely, Q1 and Q2 pockets) characterized by hydrophobic residues and responsible for the interaction of inhibitors. Specifically, Q1 is a small hydrophobic pocket pointing away from the S pocket, whereas Q2 is larger and located near Q1 [20]. The interaction between a glycine residue in the Q pocket and the cAMP purine ring was identified as critical for substrate binding. Furthermore, additional hydrogen bonds and hydrophobic interactions provide further stabilization of the PDE4/cAMP complex [21,22]. PDE4Is (e.g., Roflumilast and Rolipram, Figure 1) occupy this active site, forming a variety of interactions, such as hydrophobic interactions with conserved phenylalanine and isoleucine residues and hydrogen bonds with the invariant glutamine [22].

**Figure 2:**
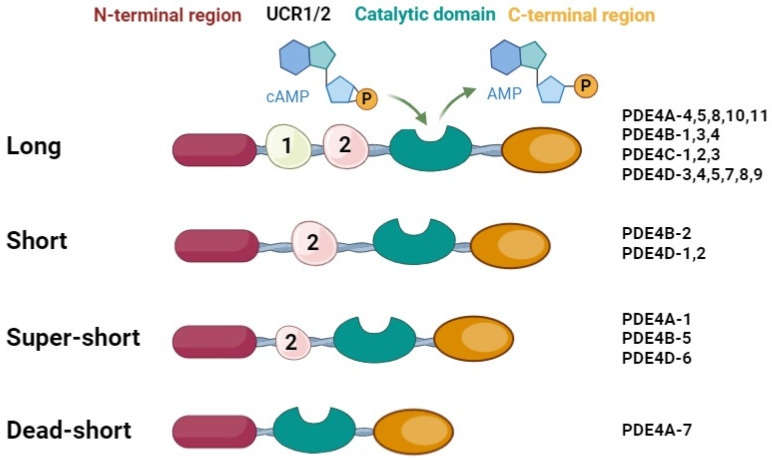
Structure of different PDE4 subtypes.

Based on the presence or absence of two upstream-conserved regions UCR1 (approximately 60-amino-acids long) and UCR2 (approximately 80-amino-acids long), located between the N-terminal and catalytic region, each PDE4 sub-group is divided into the (i) long type, containing both UCR1 and UCR2; (ii) short type, containing only the UCR2 domain; (iii) super-short type, also named ultra-short, characterized by a truncated UCR2; or (iv) dead-short type, in which both UCR1 and UCR2 are absent (Figure 2) [22,24].

The UCR domains play an important regulatory role in the PDE4 catalytic units [10]. In particular, an aromatic residue in the UCR2 domain (namely, Phe196 in PDE4D and Tyr196 in PDE4B) proved to be essential for the interaction with the substrate [25,26]. Moreover, several studies showed that enzyme dimerization can occur via UCR interactions. Dimer formation induces a conformational change in the catalytic domain, thus affecting enzyme activity [27]. In detail, dimerization is regulated by the phosphorylation of UCRs by protein kinase A (PKA) and extracellular signal-regulated kinase (ERK). Specifically, the PKA-mediated phosphorylation of a serine residue in UCR1 attenuates the ability of this domain to interact with UCR2 and increases PDE4 activity. Thus, the activity of PDE4 long forms, containing UCR1 and UCR2 domains, can be enhanced through PKA-induced phosphorylation and dimerization, whereas PDE4 short forms (lacking the UCR1 domain) cannot be regulated by PKA and only exist as a monomer [28,29,30]. In addition, the catalytic domain of all isoforms (except for PDE4A) contains a serine residue phosphorylated by ERK [31] and several other proteins (e.g., arrestin, Src family tyrosine protein kinases, receptor for activated C kinase 1 (RACK1), A-kinase-anchoring proteins (AKAPs)) that play a role in PDE4 regulation [32,33,34].

The carboxyl–terminal region contains an ERK phosphorylation site, whose phosphorylation induces the activation of PDE4 short forms and the inhibition of PDE4 long forms [35].

Recently, the conserved region 3 (CR3) domain was identified between the C-terminal region and the catalytic site of PDE4B2 and PDE4D3 isoforms. CR3 presents a conserved sequence (called FQF, Phe678-Gln679-Phe680), essential for binding with different proteins, including ERKs [26]. The presence of this additional domain could be useful for the identification of selective inhibitors, especially for the PDE4B2 and PDE4D3 isoforms [36,37].

## 2. PDE4 Inhibitors (PDEIs)

The first and most investigated PDE4I is Rolipram (Figure 1), synthesized in 1977 [38,39] and developed for psychotic disease treatment [40]; unfortunately, Rolipram and its structurally related analogs evidenced serious adverse side effects (e.g., nausea, vomiting, sedation, diarrhea, dyspepsia, and headache) in clinical evaluations [41].

In the past, these side effects have been ascribed to the ability of Rolipram to interact with two distinct PDE4 conformers called the high-affinity Rolipram binding state (HARBS) and low-affinity Rolipram binding state (LARBS) [15,42]. Thus, HARBS inhibition was involved in neurite outgrowth, myelination, and cognitive effect [43] and related to antidepressant activity and side effects such as emesis, whereas LARBS seemed to be preferentially involved in anti-inflammatory responses [44]. Consequently, PDE4Is endowed with selectivity against LARBS over HARBS could exert positive therapeutic effects with reduced emesis [45]; at the same time, the engagement with LARBS may not be appropriate for targeting CNS disorders.

In 1996, it became evident that the PDE4I side effect profile could be ameliorated by developing isoform-specific agents rather than HARBS/LARBS-selective compounds [46]. In the early 2000s, the PDE4D isoform was indicated as responsible for PDE4 inhibitor-induced emesis [47], although recent studies suggest that it may not be the only factor involved in emesis onset [48]; on the other hand, it has been demonstrated that selective PDE4BIs produce potent anti-inflammatory and reduced emetic effects [49]. Additionally, some recent selective PDE4DIs did not show emetic effects in in vivo tests on mice [50], indicating that this aspect still needs to be clarified and better investigated.

At the same time, PDE4B isoforms are highly expressed in the CNS (especially in the amygdala, thalamus, striatum, hypothalamus, and hippocampus) and immune cell system, thus playing a fundamental role in the neuroinflammation process [12]. Particularly, the PDE4B2 isoform represents 95–100% of PDE4 expressed in astrocytes, monocytes, leukocytes, and neutrophils and is involved in the control and regulation of various inflammatory stimuli, such as neutrophils accumulation, TNF-α production, LPS induction, and microglia activation.

Furthermore, elevated PDE4B levels have been experimentally evidenced in a proinflammatory phenotype in neutrophils, macrophages, and microglia. This concept was further strengthened following evidence of increased expression of PDE4B2 in experimental models of neuroinflammatory diseases [24].

### 2.1. The Pharmacological Role of the PDE4D Isoform

Several studies demonstrated the fundamental role of PDE4D isoforms in cognitive, learning, and memory consolidation processes. These roles are linked to the high expression of these isoforms in the CNS, especially in the CA1 region of the hippocampus [51]. In particular, PDE4D4 and PDE4D6 isoforms are localized exclusively at the neuronal cell level [52].

cAMP’s role in cognitive and memorization processes has been widely recognized. The increase in cAMP levels in the brain induces the activation of the cAMP response element-binding protein (CREB) by PKA, essential for synaptic plasticity and the formation of long-term memory. Conversely, a depletion of cAMP levels induced by the high expression of PDE4D subtypes D1 and D3 was observed in neurodegenerative diseases (e.g., AD) and associated with cognitive deterioration and neuroinflammation [53]. Some authors demonstrated that the inhibition of PDE4D boosts oligodendrocyte progenitor cell differentiation and, therefore, the re-myelination of axons, yielding re-myelinated shadow plaques in the CNS without inducing side effects [54]. In addition, PDE4D inhibition promoted in vivo re-myelination in a model of multiple sclerosis (MS), without triggering emesis-like side effects in rodents [55,56].

Overall, these observations strongly support the therapeutic efficacy of selective PDE4DIs for the treatment of various neurodegenerative diseases, such as AD, HD, Parkinson’s disease (PD), Lou Gehrig’s disease (ALS), stroke, traumatic brain injury (TBI), spinal cord injury (SCI), mild cognitive impairment (MCI), and demyelinating diseases such as peripheral demyelinating diseases (PNS) and MS. More recently, some selective PDE4DIs have been investigated in autism [57] and fragile X syndrome (FXS) [58]. Although the underlying mechanisms of the cognition-enhancing effect remain elusive, PDE4D inhibition appears to be an interesting novel therapeutic option for cognitive deficits, particularly in AD [59,60].

PDE4D also proved to affect the PKA-mediated phosphorylation of tau protein Serine 214. Thus, in vitro studies illustrated that the inhibition of PDE4D enhanced the Ser214 phosphorylation of tau, an event associated with early AD tau pathology. The study confirmed that age-related loss of PDE4D may contribute to the specificity vulnerability of the frontal cortex to AD degeneration, playing a critical role in normal cAMP regulation, cautioning against the use of pan-PDE4DIs as therapeutics [61].

In addition, PDE4D seems to be involved in depression, with PDE4DIs able to reverse the depression-like behaviors induced by chronic unpredictable stress (CUS) through restoring cAMP, PKA, the phosphorylation of CREB (pCREB), and GLT1 (glutamate transporter 1) levels in the hippocampus of rats [62]. This evidence supported the neuroprotective potential of PDE4DIs against CUS-induced dysfunctions and prompted Chinese researchers to patent PDEIs as potential anti-depressant agents [63]. At the same time, the PDE4D isoform has been investigated for its role in cancer development, particularly in hepatocellular carcinoma (HCC) [64], breast cancer [65], and acquired tamoxifen-resistant breast cancer [66].

PDE4D enzymes are subdivided into nine subtypes (namely, PDE4D1–9), that can be grouped into long-, short-, or super short according to the presence/length of UCR domains, as reported in Figure 2 [32]. To date, the most studied isoforms are the ones localized in the CNS system (i.e., PDE4D1, D3, D4, and D6), which are probably involved in various neurodegenerative diseases.

### 2.2. PDE4Is Approved or in Clinical Trials

Roflumilast, Apremilast, Crisaborole, and Ibudilast (Table 2) represent the four PDE4Is currently approved for clinical use. In 1989, Ibudilast was the first PDE4I to be approved for Krabbe disease, a rare childhood lysosomal disorder characterized by neurodegeneration of the white matter in the central and peripheral nervous systems [67,68]. Then, in 2010, Roflumilast (Daxas) was approved as a therapeutic agent for the treatment of chronic obstructive pulmonary disease (COPD), followed by Apremilast (Otezla) in 2014 for the treatment of psoriatic arthritis (PsA) [69,70,71]. Finally, in 2016, Crisaborole (Eucrisa) and Ibudilast were approved for atopic dermatitis and bronchial asthma, respectively [68].

Prompted by the approval of the previously mentioned PDE4Is, new PDE4Is were developed, and selected compounds reached the clinical phase. The majority of the novel inhibitors share a catechol substructure (e.g., Rolipram, one of the first synthesized and most widely studied analogs, Table 3) [22,72], but, also, pyridines (Table 3) and differently decorated bicyclic compounds (Table 4) showed relevant PDE4 inhibitory properties.

Specifically, Rolipram, belonging to the catechol family, has been evaluated in six clinical trials: two studies (a completed phase I and a recruiting phase I) concern major depressive disorder (MDD), a completed phase I study with available results is focused on depression (NCT05522673), a completed phase I for HD, and a completed phase II study for MS. Lastly, Rolipram has also undergone a now-completed phase II study (NCT02743377) in patients with McCune–Albright syndrome (MAS), a rare condition affecting bones, skin, and some hormone-producing tissues, caused by a genetic mutation that causes cAMP overproduction [73]. Unfortunately, Rolipram is characterized by relevant emetic side effects that, up to now, have prevented its commercialization.

Among Rolipram-related molecules, Tanimilast (CHF6001, Table 3) is widely studied for respiratory disorders. Indeed, this inhibitor is in two phase II studies for asthma and twelve studies (seven phase I and five phase II studies) for COPD [74,75]. Similarly, Cilomilast (SB-207499, Table 3) was investigated in a completed phase III study for COPD (NCT00103922) [75,76,77]. Difamilast (also named OPA-15406 or MM36, Table 3) has been evaluated in two phase I studies, four phase II studies (three with results), and eight phase III studies focused on atopic dermatitis. Interestingly, it is also involved in an observational study for chronic thromboembolic pulmonary hypertension (CTEPH) (NCT02114047) and a trial for joint disease (DJD) (NCT01936259) [78,79,80,81]. LEO 29102 is currently under investigation in six clinical trials for atopic dermatitis (five phase I and one phase II) and in one phase I study for psoriasis [82,83]. Finally, HT-0712 efficacy for age-associated memory impairment (AAMI) has been assessed by a completed phase II study (NCT02013310) [84,85].

**Table 3:** Structures, IUPAC names, therapeutic indications, IC_50_ values on different PDE4 isoforms, and related references of the catechol PDE4Is under clinical investigation.

Compound	Structure	Condition or Disease	IC_50_	Literature Data
Rolipram (±) 4-(3-(cyclopentyloxy)-4-methoxyphenyl)pyrrolidin-2-one	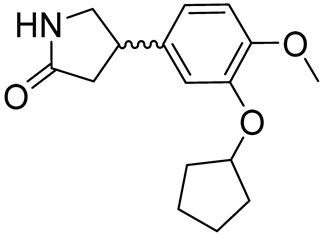	Multiple sclerosis, depression, Huntington’s disease, major depressive disorder	PDE4D = 0.24 µM	[72]
Tanimilast (CHF6001) (*S*)-3,5-dichloro-4-(2-(3-(cyclopropylmethoxy)-4-(difluoromethoxy)phenyl)-2-((3-(cyclopropylmethoxy)-4-(methylsulfonamido)benzoyl)oxy)ethyl)pyridine 1-oxide	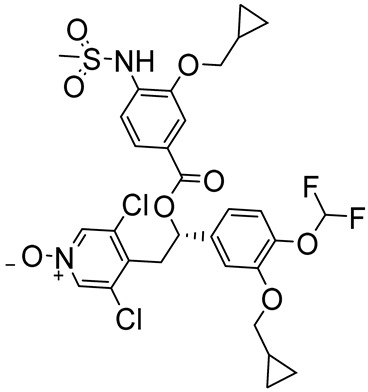	COPD, asthma	PDE4 = 26 pM	[74,75]
Cilomilast (SB-207499) 4-cyano-4-(3-(cyclopentyloxy)-4-methoxyphenyl)cyclohexane-1-carboxylic acid	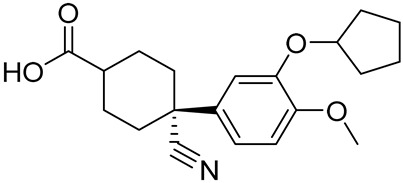	COPD	PDE4 = 120 nM	[75,76,77]
Difamilast (OPA-15406/MM36) *N*-((2-(4-(difluoromethoxy)-3-isopropoxyphenyl)oxazol-4-yl)methyl)-2-ethoxybenzamide	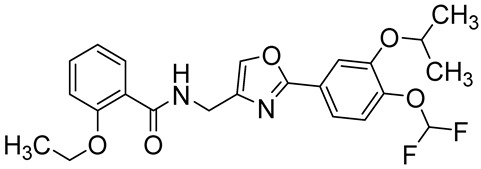	Chronic thromboembolic pulmonary hypertension, joint disease, atopic dermatitis	PDE4A = 0.0832 µM PDE4B = 0.0112 µM PDE4C = 0.2493 µM PDE4D = 0.0738 µM	[78,79,80,81]
LEO 29102 2-(6-(2-(3,5-dichloropyridin-4-yl)acetyl)-2,3-dimethoxyphenoxy)-*N*-propylacetamide	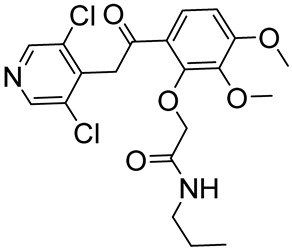	Atopic dermatitis, psoriasis	PDE4 = 5 nM	[82,83]
HT-0712 (3*S*,5*S*)-5-(3-(cyclopentyloxy)-4-methoxyphenyl)-3-(3-methylbenzyl)piperidin-2-one	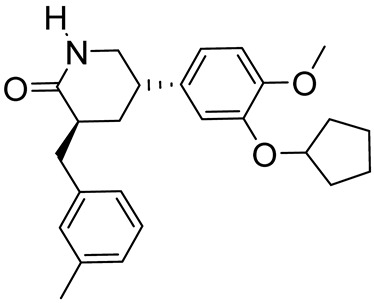	Age-associated memory impairment	PDE4D = 150 nM	[84,85]

Among pyridine derivatives, Tetomilast (OPC-6535, Table 4) is under clinical evaluation for Crohn’s disease (IBD) (one phase II and one phase III study), ulcerative colitis (SUC) (one phase II and three phase III studies), and COPD (two phase II studies) [86,87]. Similarly, a phase II clinical trial (NCT00263874) was carried out to assess the safety and efficacy of UK500,001 (Table 4) for the treatment of COPD [88]. Zatolmilast (BPN14770, Table 4) was investigated for FXS (three phase III studies), depression (phase II completed study with results, NCT03861000), and AD (three phase I and a phase II study) [89,90,91].

**Table 4:** Structures, IUPAC names, therapeutic indications, IC_50_ on different PDE4 isoforms, and related references of pyridine-based compounds in clinical trials.

Compound	Structure	Condition or Disease	IC_50_	Literature Data
Tetomilast (OPC-6535) 6-(2-(3,4-diethoxyphenyl)thiazol-4-yl)picolinic acid	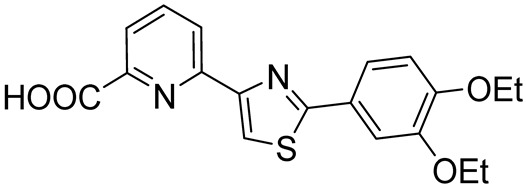	COPD, Crohn’s disease, ulcerative colitis	PDE4 = 74 nM	[86,87]
UK500,001 2-(3,4-difluorophenoxy)-5-fluoro-*N*-((1s,4s)-4-(2-hydroxy-5-methylbenzamido)cyclohexyl)nicotinamide	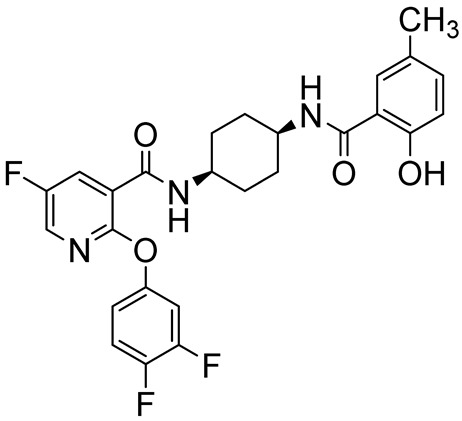	COPD	PDE4 = 0.38–1.9 nM	[88]
Zatolmilast (BPN14770) 2-(4-((2-(3-chlorophenyl)-6-(trifluoromethyl)pyridin-4-yl)methyl)phenyl)acetic acid	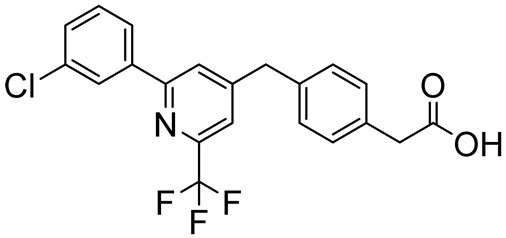	Alzheimer’s disease, fragile X syndrome, depression	PDE4D = 8 nM	[89,90,91]

Among bicyclic compounds, AWD-12-281 (GW842470X, Table 5), Orismilast (LEO-32731, Table 5), Lotamilast (E6005, Table 5), and DRM02 (Table 5) were evaluated in clinical trials for atopic dermatitis [92,93,94,95,96]. Moreover, Orismilast (four phase I and two phase II studies), DRM02 (one phase II study), MK-0873 (two completed phase I studies, Table 5), Mufemilast (Hemay005; six phase I, one phase II, and one phase III study; Table 5) have been investigated for psoriasis [13,97,98,99,100,101,102]. In addition, Orismilast was also evaluated in phase I and phase II studies for hidradenitis suppurativa (HS), whereas DRM02 was studied in a completed phase II study on rosacea. Mufemilast was also studied in a phase II trial for SUC (NCT05486104), active ankylosing spondylitis (SPA) (NCT05407246), and Behçet’s disease (BD) (NCT06145893); MK-0873 completed a phase II study (NCT00132769) on rheumatoid arthritis (RA) and terminated a phase II study on COPD (NCT00132730), both with available results. GSK256066 (Table 5) is involved in a phase I and II study for COPD, five phase II studies for rhinitis, and three phase II studies on asthma [103,104]. Conversely, GSK356278 (Table 5) is involved in one phase I trial on depressive and anxiety disorders and two phase I studies on HD [105,106]. Finally, both Etazolate and MK-0952 (Table 5) were in a completed phase II study for AD [107,108,109,110,111,112].

Interestingly, tricyclic compounds are also being studied for asthma: Oglemilast (GRC 3886, Table 5), Revamilast (Table 5), and Ensifentrine (RPL554, Table 5). Two of them, Oglemilast and Ensifentrine, are also in phase II and phase III trials for COPD, respectively. In addition, Revamilast was investigated in a completed phase II study on RA (NCT01430507), whereas Ensifentrine was investigated in a phase II study on both COVID-19 (NCT04527471) and cystic fibrosis (CF) (NCT02919995) [113,114,115,116].

**Table 5:** Structures, IUPAC names, therapeutic indications, IC_50_ on different PDE4 isoforms, and related references of bi- and tricyclic compounds in clinical trials.

Compound	Structure	Condition or Disease	IC_50_	Literature Data
AWD-12-281 (GW842470X) *N*-(3,5-dichloropyridin-4-yl)-2-(1-(4-fluorobenzyl)-5-hydroxy-1*H*-indol-3-yl)-2-oxoacetamide	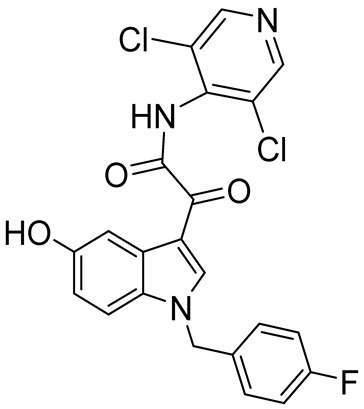	Atopic dermatitis	PDE4 = 9.7 nM	[92,93]
Orismilast (LEO-32731) 3,5-dichloro-4-(2-(4-(difluoromethoxy)-1′,1′-dioxido-2′,3′,5′,6′-tetrahydrospiro[benzo[d][1,3]dioxole-2,4′-thiopyran]-7-yl)-2-oxoethyl)pyridine 1-oxide	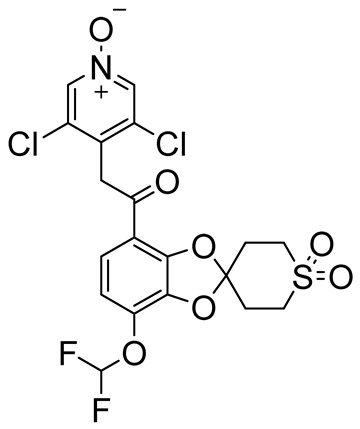	Psoriasis, atopic dermatitis, hidradenitis suppurativa	PDE4B = 6–16 nM PDE4D = 3–9 nM	[13,97]
Lotamilast (E6005) ethyl 4-((3-(6,7-dimethoxy-2-(methylamino)quinazolin-4-yl)phenyl)carbamoyl)benzoate	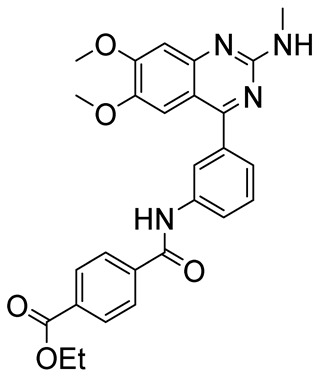	Atopic dermatitis	PDE4 = 2.8 nM	[94,95,96]
DRM02 2-(5-amino-3-methyl-1H-pyrazol-4-yl)-5-fluoro-N-methylbenzo[d]thiazole-6-sulfonamide	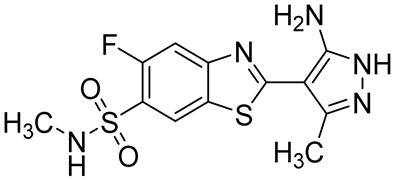	Rosacea, atopic dermatitis, psoriasis	PDE4A = 0.64 µM PDE4B = 0.44 µM PDE4D = 0.63 µM	[98]
MK-0873 3-((3-(3-(cyclopropylcarbamoyl)-4-oxo-1,8-naphthyridin-1(4H)-yl)phenyl)ethynyl)pyridine 1-oxide	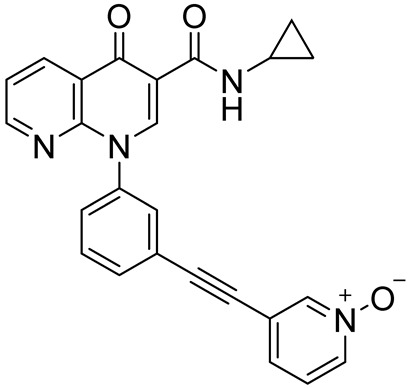	Rheumatoid arthritis, psoriasis, COPD	PDE4 = 38 nM	[99,100]
Mufemilast (Hemay005) (*S*)-N-(5-(1-(3-ethoxy-4-methoxyphenyl)-2-(methylsulfonyl)ethyl)-4,6-dioxo-5,6-dihydro-4*H*-thieno[3,4-*c*]pyrrol-1-yl)acetamide	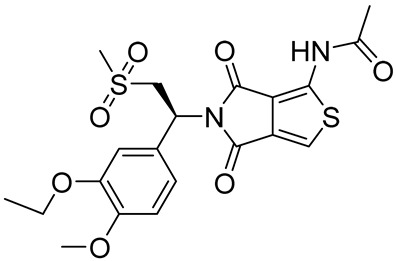	Psoriasis, severe ulcerative colitis, Behçet’s disease, active ankylosing spondylitis	PDE4 = 80–120 nM	[101,102]
GSK256066 6-((3-(dimethylcarbamoyl)phenyl)sulfonyl)-4-((3-methoxyphenyl)amino)-8-methylquinoline-3-carboxamide	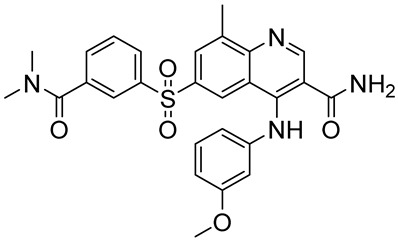	COPD, rhinitis, asthma	PDE4 = 3.2 pM	[103,104]
GSK356278 5-(5-((2,4-dimethyl-4,5-dihydrothiazol-5-yl)methyl)-1,3,4-oxadiazol-2-yl)-1-ethyl-*N*-(tetrahydro-2H-pyran-4-yl)-1*H*-pyrazolo[3,4-*b*]pyridin-4-amine	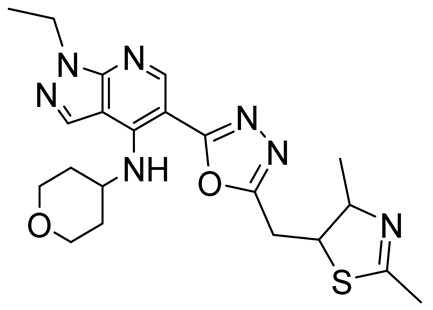	Depressive and anxiety disorders, Huntington’s disease	PDE4A = 2.51 nM PDE4B = 1.58 nM PDE4D = 2.00 nM	[105,106]
Etazolate ethyl 1-ethyl-4-(2-(propan-2-ylidene)hydrazineyl)-1*H*-pyrazolo[3,4-*b*]pyridine-5-carboxylate	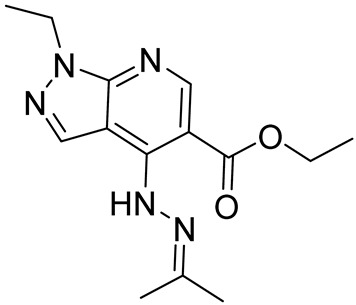	Alzheimer’s disease	PDE4 = 2 µM	[107,108,109,110]
MK-0952 (1*S*,2*S*)-2-(3′-(3-(cyclopropylcarbamoyl)-4-oxo-1,8-naphthyridin-1(4*H*)-yl)-3-fluoro-[1,1′-biphenyl]-4-yl)cyclopropane-1-carboxylic acid	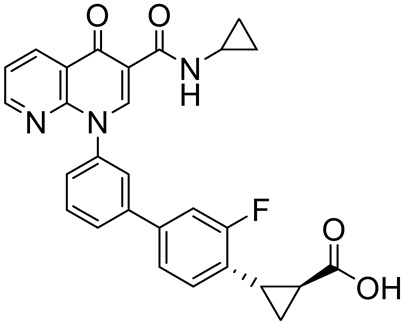	Alzheimer’s disease	PDE4 = 0.6 nM	[111,112]
Oglemilast (GRC 3886) *N*-(3,5-dichloropyridin-4-yl)-4-(difluoromethoxy)-8-(methylsulfonamido)dibenzo[b,d]furan-1-carboxamide	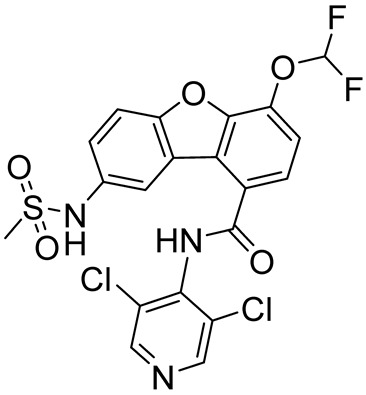	COPD, asthma	PDE4 = 0.5 nM	[113,116]
Revamilast 3,5-dichloro-4-(6-(difluoromethoxy)benzofuro[3,2-*c*]pyridine-9-carboxamido)pyridine 1-oxide	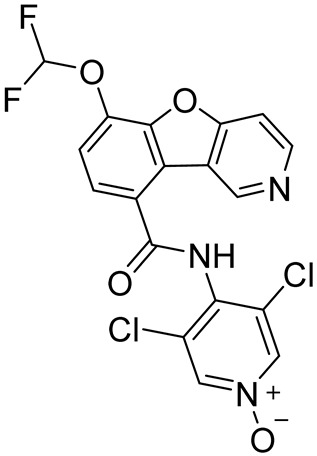	Asthma, rheumatoid arthritis	PDE4 = 2.7 nM	[114,116]
Ensifentrine (RPL554) (*E*)-1-(2-(2-(mesitylimino)-9,10-dimethoxy-4-oxo-6,7-dihydro-2*H*-pyrimido[6,1-a]isoquinolin-3(4*H*)-yl)ethyl)urea	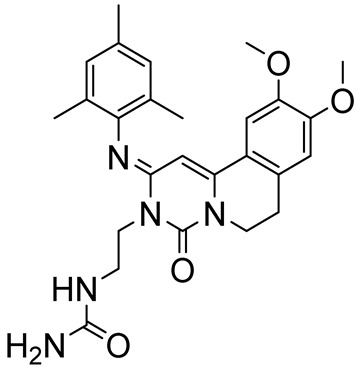	COPD, asthma, COVID-19, cystic fibrosis	PDE4 = 1.48 µM	[115]

## 3. PDE4D Inhibitors (PDE4DIs)

The high expression levels of PDE4D isoforms in the CNS support medicinal chemistry studies for the identification of selective PDE4Dis, useful for the treatment of different cognitive disorders. Based on their chemical structures, the PDE4DIs so far reported in the literature can be classified as (i) catechol derivatives, (ii) pyridine and pyrimidine compounds, (iii) quinoline-based derivatives, (iv) pyridazinones and naphthyridines, and (v) miscellaneous and natural compounds. The identified compounds can act either as catalytic inhibitors or allosteric modulators, as detailed below [48].

### 3.1. Catechol-Based Compounds

The catechol ether scaffold is commonly embedded in the structure of PDE4Is, sharing with the adenine cAMP portion a similar hydrophobic nature [22]. The X-ray crystal structures of different PDE4D/PDE4DI complexes showed that the catechol moiety forms key interactions with invariant glutamine (hydrogen bonds) and phenylalanine (π-π stacking interaction) residues, with these contacts being essential for enzyme affinity [117]. As previously mentioned, Roflumilast (Table 2) was the first marked PDE4DI with IC_50_ values of 0.68 nM [118,119,120,121], while compounds HT-072 (PDE4D IC_50_ = 150 nM), Difamilast (PDE4D IC_50_ = 73.8 nM), and LEO29102 (PDE4D IC_50_ = 5 nM) are currently in clinical trials for the treatment of atopic dermatitis, psoriasis, and AAMI, as reported in Table 3 [81,83,85].

**Figure 3:**
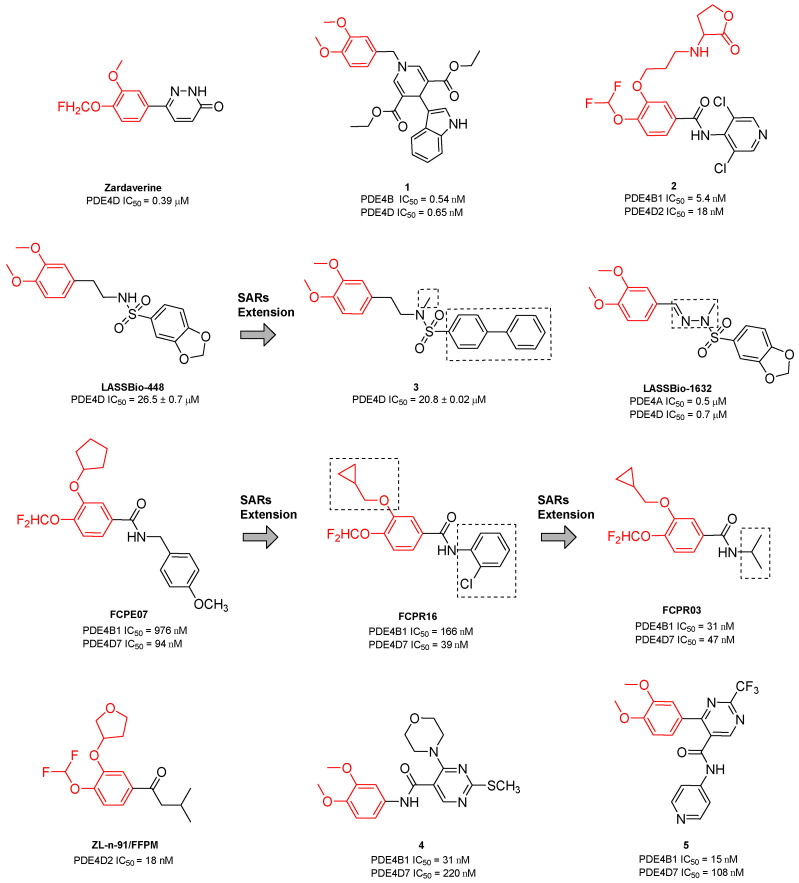
Catechol-based PDE4DIs. The catechol portion is highlighted in red. Structural modifications of lead compounds LASSBio-448 and FCPE07 are reported in boxes.

Zardaverine (Figure 3) was synthesized in 1984 and displayed dual PDE inhibitory activity with equal potency against PDE3 and PDE4 [122]. In particular, the compound seemed to specifically inhibit the PDE4D isoform with an IC_50_ of 0.39 µM and proved to block in vivo bronchoconstriction 100-fold more potently than theophylline. The molecule reduced bronchial eosinophilia and airway hyperactivity (AHR) in guinea pigs and ameliorated the airway function of patients with chronic airway obstruction by interfering with TNF formation [122,123,124]. The efficacy of inhaled/instilled and oral Zardaverine was demonstrated in guinea pigs and the prerequisites for human studies were provided by oral and inhalation toxicology. Unfortunately, adverse effects involving the CNS, especially vomiting, discouraged further development for oral administration. In inhalation studies, Zardaverine was well tolerated, but the development of the drug was discontinued due to its fast elimination [122]. More recently, the compound showed selective in vitro and in vivo antitumor activity against HCC, unrelated to PDE inhibition [125].

In 2013, Poondra and collaborators synthesized a series of chatecol-based 1,4-dihydropyridine compounds able to interact with PDE. In detail, derivative **1** (Figure 3) displayed the highest inhibitory activity against PDE4B (IC_50_ = 0.54 nM) and PDE4D (IC_50_ = 0.65 nM) and was able to block the release of LPS-induced TNF-α with an IC_50_ of 3.20 ± 0.53 µM. Docking simulation on the PDE4D/**1** complex showed a possible interaction between the dimethoxy group of the catechol moiety and Gln443 and between the indole and Glu505, Glu396, and Ser442 [126].

One year later, Boland and coworkers isolated a series of Roflumilast analogs as soft PDEIs. Among the obtained compounds, analog **2** (Figure 3) displayed the best selectivity for PDE4D with an IC_50_ of 18 nM. The molecule showed a good permeability profile in a parallel artificial membrane permeability assay (PAMPA) but a very fast degradation in a preliminary stability evaluation [127].

In 2016, Nunes’s group designed and synthesized as anti-inflammatory agents a series of sulfonamides bearing a catechol moiety. Among the isolated derivatives, LASSBio-448 (Figure 3) displayed high selectivity for the PDE4D isoform, with an observed IC_50_ of 26.5 µM. The compound proved to inhibit allergen- or LPS-induced lung inflammation and aryl hydrocarbon receptors in mice, blocking the cascade of pro-inflammatory mediators such as IL-4, IL-5, IL-13, and eotaxin-2. Furthermore, the lower pro-emetic effect in comparison with Rolipram and Cilomilast highlighted its marked potential for the treatment of lung inflammation diseases such as asthma [128]. Further modification on the aryl substituent of the sulphonyl amide and the introduction of a sulfonyl hydrazone as a linker to the catechol group led to the identification of another series of derivatives, which were evaluated on several PDE4 isoforms. Compounds **3** (PDE4D IC_50_ = 20.8 µM, Figure 3) and LASSBio-1632 (PDE4D IC_50_ = 0.7 µM, Figure 3) displayed a selective effect on PDE4A and PDE4D and were selected for in vivo evaluations. Although **3** did not exhibit significant inhibition of LPS-induced inflammation, LASSBio-1632 proved to inhibit the release of TNF-α in the lung tissue in a dose-dependent fashion, being 8-fold more effective than the reference compound LASSBio-448. The novel molecule was also able to relax guinea pig trachea on non-sensitized and sensitized animals, displaying great gastrointestinal permeability [129].

Amide FCPE07 (Figure 3), synthesized by Zhou and colleagues, displayed comparable PDE4 inhibitory activity with Rolipram and exhibited a 10-fold selectivity for PDE4D (IC_50_ = 94 nM) over the PDE4B subtypes and more than 1000-fold selectivity against other PDE family members [130]. The simplification of the amide substituent and the replacement of the cyclopentyl group of the catechol moiety with a cyclopropylmethyl function led to compound FCPR16 (Figure 3), which maintained good activity and selectivity for the PDE4D isoform (IC_50_ = 39 nM). FCPR16 exhibited anti-neuroinflammation potential by inhibiting LPS-induced TNF-α production in microglia. According to docking simulations, FCPR16 and Roflumilast would adopt similar binding conformations within PDE4 purine-selective glutamine and hydrophobic clamp pockets (i.e., Q1 and Q2 regions) [131].

Finally, the introduction of a shorter *N*-alkyl side chain allowed for the identification of compound FCPR03 (Figure 3), which displayed nanomolar inhibitory activities against PDE4B (IC_50_ = 31 nM) and PDE4D (IC_50_ = 47 nM). The derivative was able to interfere with TNF-α, inducible nitric oxide synthase (iNOS), and cyclooxygenase-2 (COX-2) production in microglia and, interestingly, exhibited antidepressant effects in tail suspension and forced swim tests, without causing emesis [132]. Further investigation suggested that FCPR03 has therapeutic potential in preventing and treating cerebral ischemia, possibly through the upregulation of the AKT/GSK3β/β-catenin signaling pathway. In fact, the molecule suppressed reactive oxygen species (ROS) generation and restored the function of mitochondria in neuronal cells following oxygen–glucose deprivation insult. Additionally, FCPR03 enhanced AKT and GSK3β phosphorylation and increased Epac2 and β-catenin levels in the brain tissues of rats subjected to middle cerebral artery occlusion followed by reperfusion [133].

In 2017, Guo’s group investigated the anti-inflammatory properties of ZL-n-91 (Figure 3), a selective PDE4DI (IC_50_ = 18 nM) synthesized by Zheng and collaborators [134,135]. The compound was able to reverse learning and memory impairments in a transgenic mouse model of AD and restored cAMP, p-PKA, p-CREB, and brain-derived neurotrophic factor levels in the hippocampus of APP/PS1 transgenic mice. Furthermore, ZL-n-91 exerted anti-inflammatory effects through the reduction of the release of NF-kB and cytokines (e.g., TNF-α and IL-1β). Interestingly, this simple isovaleryl ketone showed a low emetic potency in mice compared to the vehicle treatment [135]. In further studies, the catechol molecule displayed antitumor activity against MDA-MB-231 and BT-549 cells, without affecting the proliferation of normal human keratinocytes (HaCaT cells). The selective cytotoxic effect on tumor cells was probably due to its interaction with cell cycle-related proteins, such as cyclin-dependent kinase (CDK) 2, CDK4, cyclin D1, PCNA, and p-RB. Additionally, ZL-n-91 prevented the growth of the transplanted MDA-MB-231 tumor xenograft in nude mice and increased γ-H2AX expression, highlighting its potential use in anticancer therapy [136].

Finally, Purushothaman and coworkers synthesized a series of catechol–pyrimidine PDE4Is for the treatment of atopic dermatitis. Derivatives **4** and **5** (Figure 3) displayed good selectivity for PDE4B (IC_50_ = 31 nM and 15 nM, respectively) and PDE4D (IC_50_ = 220 nM and 108 nM, respectively) and were selected for preliminary in vivo investigations in mice [137]. Compound **5**, bearing the catechol portion directly bonded to the pyrimidine core, displayed remarkable anti-inflammatory activity by blocking the release of pro-inflammatory cytokines (e.g., TNF-α, IL-4, IL-5, and IL-17) and reducing infiltrative CD4+ T-helper cells, mast cells, and IgE levels in atopic tissue [138].

**Figure 4:**
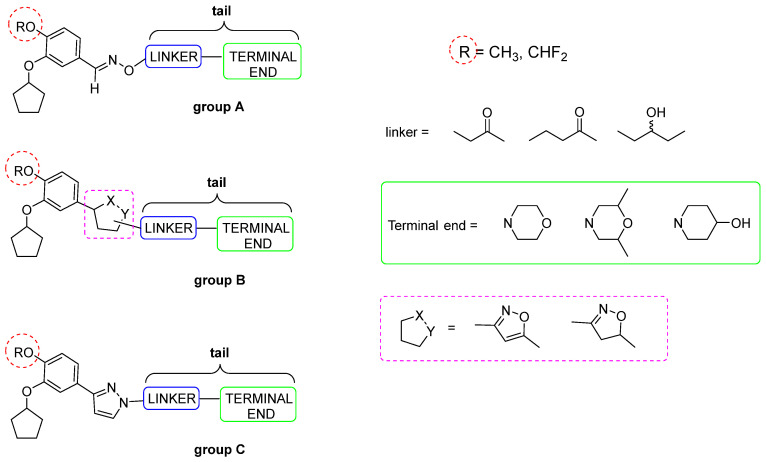
General structure of the GEBR library (groups A, B, and C).

At the University of Genova (Italy), a large library of catechol-derived molecules (200 molecules, named the GEBR library) has been synthesized to obtain selective PDE4DIs. In detail, in these compounds, the catechol scaffold is linked to a terminal amine or amide group through different linkers including the (i) imino-ether function (group A, Figure 4); (ii) isoxazole or isoxazoline ring, in which the imino-ether chain is cyclized and less flexible (group B, Figure 4); and (iii) pyrazole core, directly linked to the catechol portion (group C, Figure 4).

**Figure 5:**
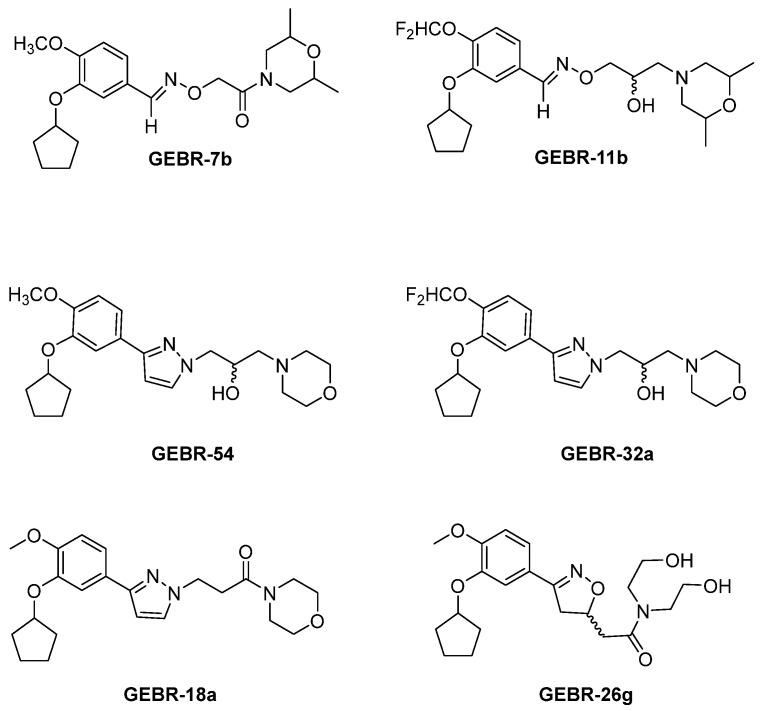
Molecular structure of catechol-based PDE4DIs **GEBR-7b**, **GEBR-11b**, **GEBR-54**, **GEBR-32a**, **GEBR-18a**, and **GEBR-26g**.

Group B compounds proved to be poorly active against PDE4D and PDE4B isoforms, while **GEBR-7b**, **GEBR-11b** (group A, Figure 5), **GEBR-54** (group C, Figure 5), and its fluorinated analog **GEBR-32a** (group C, Figure 5) presented good enzymatic profile and interesting in vivo activity. In detail, these four compounds showed IC_50_ values on PDE4D3 of 1.91 μM, 0.19 μM, 4.6 μM, and 1.0 μM, respectively, with a good selectivity profile; furthermore, they did not show genotoxic or cytotoxic effects, and **GEBR-54** and **GEBR-32a** were well absorbed and quickly distributed in the brain [139,140,141,142].

**GEBR-7b** evidenced some interesting pharmacological properties, being able to increase the extracellular cAMP levels in the hippocampus (micro dialysis assay on moving mice) and avoid the accumulation of β-amyloid in vivo, being 10 times more effective than Rolipram in AD animal models (Wistar rats and C57B16/6NCrl mice). Furthermore, the compound was found to be ten times more active than Rolipram in improving learning and memory (“object location test” and “object recognition test”) and, unlike Rolipram and its analogs, it caused an emetic effect only at doses 100 times higher than the effective dose [50,59,143,144].

It has also been observed that **GEBR-32a** (dose of 100 μM) increased cAMP concentrations in HTLA neuronal cells and rat hippocampal slices in vivo. In the “object location” and “object recognition” experiments, **GEBR-32a** improved the memory and cognitive abilities of both “wild type” and Tg2576 mice. Furthermore, unlike many other PDE4Is, it did not cause emetic or sedative effects [145]. In addition, **GEBR-32a** showed good pharmacokinetic parameters (e.g., plasma AUC_0-t_ ratio = 2.71, indicating a favorable brain penetration) that further support the patent application for the **GEBR-32a** compound [146,147].

Crystallographic studies evidenced that the stabilization of the substituted catechol nucleus in the catalytic site is mainly due to the formation of hydrophobic interactions with two amino acid residues located in the Q pocket (i.e., Ile502 and Phe538) [148]. Furthermore, according to the orientation of the tails of the different compounds, the binding conformations of the GEBR library can be divided into three different groups: (i) “protruding” compounds (**GEBR-7b**, **GEBR-11b**, **GEBR-54**, and **GEBR-32a**), in which the tail extends outside the active site, occupying the S pocket and forming water-mediated contacts with outermost amino acid residues; (ii) “twisted” compounds (such as pyrazole derivative **GEBR-18a**, Figure 5), in which the tail rotates and creates intramolecular interactions that allow the terminal residue to be arranged in parallel above the pyrazole ring; and (iii) “extended” compounds (such as **GEBR-26g**, Figure 5), in which the tail is positioned unrotated and flattened on the active site, between the M and S pockets and oxygen and nitrogen atoms of the isooxazoline ring, facing towards the outside of the pocket, without forming further interactions.

Noteworthily, the most active compounds of the GEBR library (e.g., **GEBR-7b**, **GEBR-54**, and **GEBR-32a**) assumed a “protruding” bioactive conformation, thus pointing to this structural feature as a key determinant for the further development of the library. Moreover, the length and flexibility affected the selectivity profile of the compounds, and the derivatives with shorter linkers (e.g., **GEBR-7b**) were less selective towards PDE4D than those with a longer and more flexible tail (e.g., **GEBR-32a**). This difference in selectivity is ascribable to the ability of the flexible compounds to interact with the UCR2 regulatory domain of the long PDE4 isoform (Figure 1).

These investigations have also shown that the presence of the difluoro methoxy group in the catechol portion is beneficial for activity, with the fluorine atoms involved in hydrophobic interactions with the amino acid residues inside the Q1 pocket, further stabilizing the complex [148].

### 3.2. Pyridine- and Pyrimidine-Based PDE4DIs

Pyridine and pyrimidine substructures are shared by several PDE4Is, as confirmed by the wide variety of compounds reported in the literature or currently in clinical trials [15,121,149]. The pyridine-based Zatomilast (Table 3) proved to be a negative allosteric modulator of the PDE4D enzyme (IC_50_ = 8 nM) and has been evaluated in two clinical trials for the treatment of AD and FXS [91]. Moreover, the pyridine-1-oxide Orismilast (Table 4) is a second-generation small-molecule PDE4I currently in clinical development for the resolution of chronic inflammatory skin diseases. The compound displayed good selectivity for PDE4B (IC_50_ = 6–16 nM) and PDE4D (IC_50_ = 3–9 nM) isoforms, being a potential therapeutic agent for psoriasis and atopic dermatitis [97].

**Figure 6:**
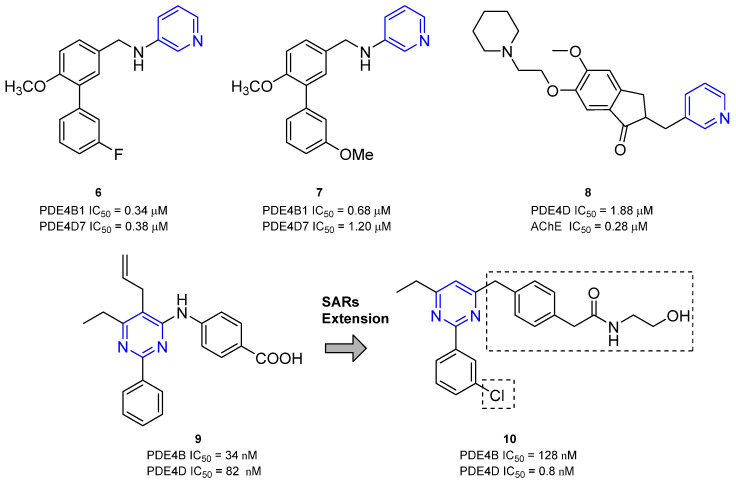
PDE4DIs with pyridine and pyrimidine scaffolds. The pyridine and pyrimidine portions are colored in blue. Structural modifications of lead compound **9** are reported in boxes.

Taking as lead compounds FCPR03 and FCPR16 (Figure 3), Tang and coworkers designed and synthesized a novel class of arylbenzylamines as PDE4Is. Derivatives **6** and **7** (Figure 6), bearing a pyridin-3-amine side chain, displayed significant inhibitory activities against human PDE4B1 (IC_50_ = 0.34 µM and 0.68 µM, respectively) and PDE4D7 (IC_50_ = 0.38 µM and 1.20 µM, respectively) and proved to bind the UCR2 region in preliminary docking simulations. The two compounds did not exhibit any cytotoxicity against SY5Y cells and showed neuroprotective effects on MPP^+^-induced apoptosis in SY5Y cells. Finally, **6** displayed higher oral bioavailability than the lead FCPR03, highlighting its pharmaceutical attractiveness [150].

Since both PDE4 and acetylcholinesterase (AChE) modulators led to improvements in cognitive and memory function, Liu and coworkers designed and synthesized a series of dual PDE4/AChE inhibitors to treat AD. Among the series, compound **8** (Figure 6), characterized by a pyridine-3-yl substituent, significantly inhibited AChE (IC_50_ = 0.28 µM) and PDE4D (IC_50_ = 1.88 µM) enzymes and exhibited low neurotoxicity and good neuroprotective effects on Aβ25–35-induced PC12 cell death. Moreover, **8** revealed comparable AChE inhibition activity with Donepezil in the brain of AD model mice, but stronger anti-neuroinflammation properties [151].

In 2009, Naganuma and coworkers reported a library of 2-arylpyrimidine derivatives as dual PDE4B/PDE4DIs with good anti-inflammatory properties. Although the novel molecules proved to be more selective for PDE4B, compound **9** (Figure 6) displayed good inhibitory activity against both the isoforms, with IC_50_ values of 34 nM and 82 nM for PDE4B and PDE4D, respectively [49]. The modification of the amino substituent bonded to the pyrimidine core made by Gurney and collaborators led to derivative **10** (Figure 6), which exhibited higher selectivity for PDE4D over PDE4B (IC_50_ = 0.8 nM versus 128 nM). The molecule showed improved pharmacokinetics properties, and the selectivity for the activated PDE4D dimeric form provided potent memory-enhancing effects, with improved tolerability and reduced vascular toxicity over earlier PDE4Is that lacked subtype selectivity [90,152].

### 3.3. Quinoline-Based PDE4DIs

Quinoline-based compounds have been widely studied for their inhibitory activities on PDE4 isoforms, as confirmed by compound GSK256066 (Table 4), currently in clinical trials for the treatment of pulmonary inflammatory diseases such as COPD, rhinitis, and asthma [104].

**Figure 7:**
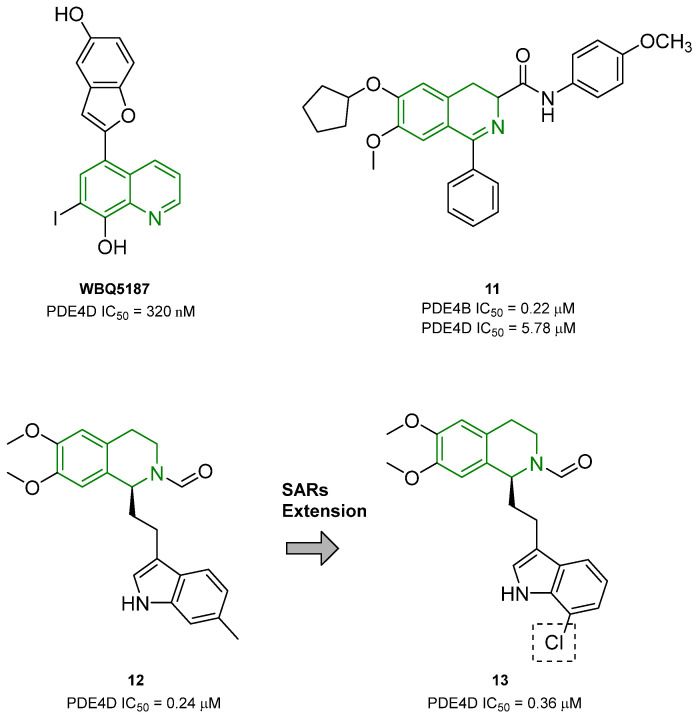
Quinoline-based PDE4DIs. The quinoline portion is highlighted in green. The structural modification of lead compound **12** is reported in the box.

In 2015, Wang and coworkers designed and synthesized a series of quinolone–benzofuran derivatives as multitargeted anti-AD compounds. Preliminary results demonstrated that these hybrid derivatives possessed significant inhibitory activities against PDE4D and abnormal β-amyloid (Aβ) aggregation, as well as remarkable antioxidant effects and excellent blood–brain barrier permeability. In particular, WBQ5187 (Figure 7) was identified as the most promising compound (PDE4D IC_50_ = 0.32 µM), with significant antioxidant effects and appropriate bio-metal chelating functions. Interestingly, the molecule modulated metal-induced Aβ aggregation and distinctively disaggregated self- or metal-induced Aβ aggregates. In addition, in AD model rats, an oral intake of 30 mg/kg/day of WBQ5187 significantly improved their spatial memory and preserved hippocampal neurons without cytotoxicity [153]. The pharmacokinetics and therapeutic efficacy of the compound were further investigated in an APP/PS_1_ mouse model of AD. WBQ5187 displayed proper oral bioavailability, metabolic stability, and excellent blood–brain barrier (BBB) permeability. Furthermore, a dose of 40 mg/kg of WBQ5187 in a 12-week treatment was able to enhance the learning memory performance of the APP/PS_1_ mice, with a more potent effect than the reference clioquinol. Additionally, the cerebral β-amyloid pathology, as well as gliosis and neuronal cell loss, were notably reduced in the mice treated with the molecule, and a cAMP increase in the hippocampus was observed [154].

Taking advantage of a structure-based drug design and fragment identification strategy, Liao and coworkers prepared novel selective PDE4Is bearing the 1-phenyl-3,4-dihydroisoquinoline scaffold. Compound **11** (Figure 7), characterized by a quinoline core substituted with methoxy and cyclopentyloxy groups, displayed high selectivity for the PDE4B and PDE4D isoforms, with an IC_50_ of 0.22 µM and 5.28 µM, respectively. The derivative seemed to attenuate inflammation by blocking the release of LPS-induced TNF-α in human blood, with comparable efficacy to Rolipram [155].

In the same year, Zhang and coworkers isolated a series of tetrahydro-isoquinoline derivatives as PDE4Is endowed with anti-inflammatory properties. Compound **12** (Figure 7) selectively inhibited PDE4D (IC_50_ = 0.24 µM) and was able to block TNF-α production in hPBMC cells. The molecule easily penetrated into the cells and showed a good safety profile, characterized by a low inhibition of dopamine receptors or hERG in comparison with the lead compound berberine. Furthermore, **12** significantly prevented LPS-induced inflammation and IMQ-induced psoriasis-like skin lesions in mice [156].

**Figure 8:**
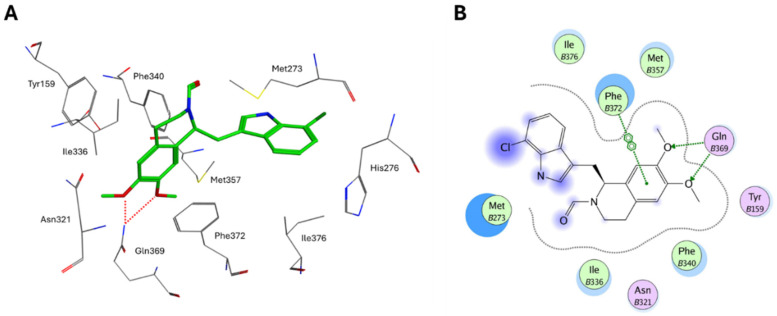
(**A**) Binding mode of compound **13** within PDE4D binding site (PDB code: 7CBJ) [157]. H-bonds are reported as red dotted lines. (**B**) Ligplot representation of receptor/ligand interactions.

Finally, the replacement of 6-methylindolyl moiety of **12** with a 7-chloroindolyl substructure led to the identification of derivative **13** (Figure 7), which displayed the same selectivity for PDE4D (IC_50_ = 0.24 µM) but improved inhibition of LPS-induced TNF-α production in RAW264.7 and hPBMC cells. The novel compound showed a good safety and permeability profile and, in preliminary in vivo studies, displayed superior therapeutic effects than the reference calcipotriol against the imiquimod (IMQ)-induced murine psoriasis-like skin inflammation after topical administration [157]. According to X-ray crystallographic studies, the PDE4D/**13** complex (PDB code: 7CBJ) is mainly stabilized by two H-bonds between the methoxy groups and Gln369 and a π-π stacking interaction between Phe372 and the tetrahydroisoquinoline scaffold (Figure 8).

### 3.4. Pyridazinone- and Naphthyridine-Based PDE4DIs

**Figure 9:**
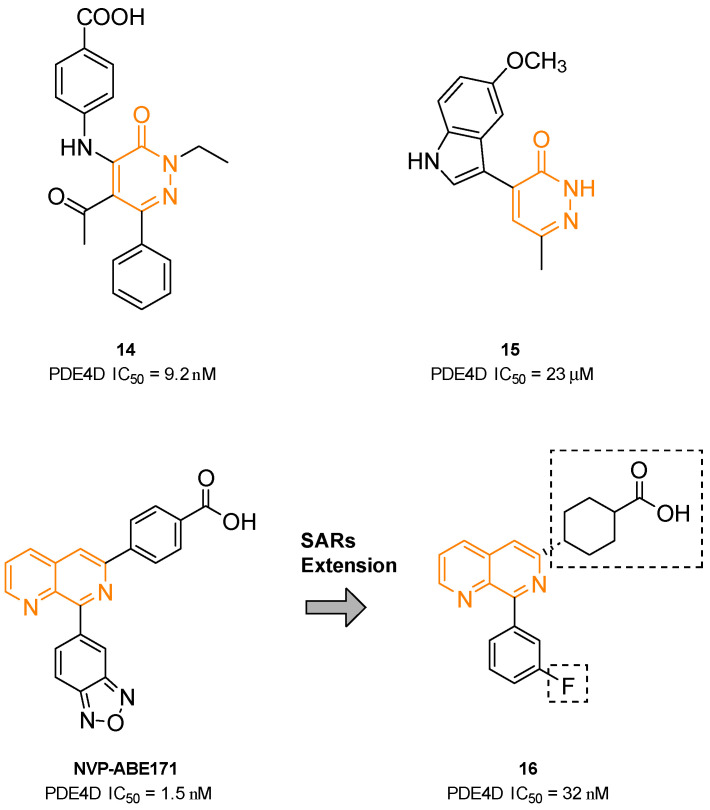
PDE4DIs with pyridazinone and naphthyridine scaffolds. The pyridazinone and naphthyridine portions are colored orange. Structural modifications of lead compound NVP-ABE171 are reported in boxes.

Due to its ability to bind the hydrophobic clamp region of the PDE4 catalytic site, the pyridazinone scaffold represents a privileged substructure for the preparation of PDE4Is [121,158]. Thus, compound **14** (Figure 9) exhibited strong PDE4D inhibition (IC_50_ = 9.2 nM) and proved to inhibit the neutrophils’ increase in the bronchoalveolar lavage fluid in an LPS-induced neutrophils rat model. As assessed by X-ray crystallography, the 2-ethyl substituent of the derivative fit into the small Q1 lipophilic pocket, whereas the phenyl ring in position six occupied the large Q2 pocket [158,159].

In 2018, Barberot and collaborators identified two families of pyridazinone derivatives as potential PDE4Is and evaluated their anti-inflammatory properties. Compound **15** (Figure 9) bore 5′-methoxyindole at position four and exhibited good PDE4 selectivity versus PDE1, with an IC_50_ of 23 nM. The new derivative did not display any cytotoxic or abnormal pro-inflammatory effect in an LDH assay and was able to block IL-8 release [160].

The naphthyridine substructure proved to be able to bind the hydrophobic clamp of the enzyme and form a hydrogen bond and π-π interaction with various amino acids within the pocket; for this reason, a number of PDE4Is are characterized by the naphthyridine heterocycle [118]. In particular, the 6,8-disubstituted 1,7-naphthyridine derivative NVP-ABE171 (Figure 9) displayed potent and selective PDE4D inhibition (IC_50_ = 1.5 nM) and induced a significant reduction in TNF-α release. A preliminary evaluation of rat model adjuvant-induced arthritis demonstrated a dose-dependent anti-inflammatory effect, without any cytotoxic evidence. Unfortunately, the poor solubility and pharmacokinetics properties precluded further investigation in humans [161].

To improve the pharmacokinetic profile of NVP-ABE171, Press and colleagues recently prepared derivative **16** (Figure 9). In detail, the benzoic acid moiety was replaced by a cyclohexyl group, while the oxadiazole ring was substituted by an *m*-fluorophenyl portion. Despite **16** showing reduced inhibition potency against PDE4D (IC_50_ = 32 nM), it exhibited a better solubility profile and reduced emetic effect, still maintaining its significant anti-inflammatory activity in an LPS-induced lung inflammation model in mice [162]. Further investigation highlighted that **16** enabled a novel escalating dosing regimen, without showing any significant nausea or emesis effect, even at high plasma levels of the drug. However, the mechanism of action of the molecule and its interaction with the PDE4 enzyme is still under investigation [163].

### 3.5. Miscellaneous PDE4DIs

**Figure 10:**
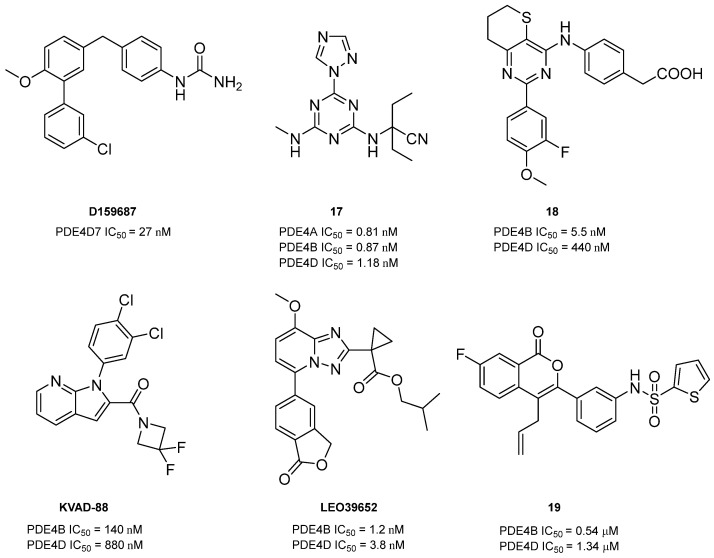
Other heterocyclic PDE4DIs reported in the literature.

Among the PDE4Is currently in clinical trials, GSK356279 (PDE4D IC_50_ = 2 nM, Table 5) and DRM02 (PDE4D IC_50_ = 0.63 µM, Table 5) displayed potent effects on the PDE4D isoform. The first compound showed excellent anti-inflammatory properties against pulmonary diseases such as COPD, rhinitis, and asthma, while the second derivative exhibited interesting potential as anti-psoriasis and other correlated inflammatory pathologies agents [98,106]. As previously discussed, the first PDE4Is explored in human clinical trials were catalytic site inhibitors, blocking the enzyme activity at high concentrations. Although this traditional approach has demonstrated therapeutic benefits, competitive inhibitors are likely to alter cAMP concentrations beyond normal physiological levels, leading to side effects such as emesis and diarrhea [164]. To identify alternative strategies to target PDE4D, in 2008, Burgin’s group developed a series of novel PDE4 modulators by a structure-based approach. Derivative D159687 (PDE4D7 IC_50_ = 27 nM, Figure 10) selectively inhibited the PDE4D isoform and was further investigated in rodent cognition assays [48]. The compound was completely distributed in the mouse brain after intravenous administration and showed a dose–response curve similar to that of Rolipram. This allosteric modulator provided cognitive benefit when administered orally and displayed substantially wider therapeutic windows with respect to emesis than the PDE4 full inhibitors [48]. Several years later, Muo and collaborators further exploited the biological properties of D159687 by studying the fat mass loss in mice treated with the compound. The PDE4 allosteric modulator seemed to induce weight loss in aged rodents, without interfering with cognitive and physical functions. Unfortunately, the higher mortality rate in the treated mice limited its potential effect [165]. More recently, Jino and coworkers assessed that D159687 influenced hippocampal cAMP levels, enhancing memory formation and consolidation and ameliorating working memory deficits in the Y-maze test, without causing sedation. Importantly, an emetic-like effect was observed only at a high dose (30 mg/kg) in a ketamine–xylazine-induced anesthesia model [166].

In 2013, Gewald and collaborators discovered a novel series of triazine derivatives as orally active PDE4Is. Compound **17** (Figure 10) displayed potent inhibitory activity against PDE4A (IC_50_ = 0.81 nM), PDE4B (IC_50_ = 0.87 nM), and PDE4D (IC_50_ = 1.18 nM). Moreover, **17** showed interesting effects on LPS-induced neutrophil accumulation, good water solubility, and acceptable pharmacokinetics parameters in preliminary in vitro evaluations [167].

In the same year, Suzuki and coworkers reported the thiopyrano[3,2-*d*]pyrimidinyl compound **18** (Figure 10) as a potent and selective PDE4B/PDE4D inhibitor with an observed IC_50_ of 5.5 nM and 440 nM, respectively. The derivative significantly interfered with TNF-α production in cell-based assays but displayed a weaker effect than Roflumilast on LPS-induced pulmonary neutrophilia in mice [168].

Taking into consideration that chronic exposure to cocaine elevates cAMP levels as a neuroadaptive response in reward-related brain regions, Burkovetskaya’s group synthesized a new PDE4I with potential clinical use for cocaine addiction. Particularly, derivative KVAD-88 (Figure 10) proved to significantly inhibit PDE4B (IC_50_ = 140 nM) and PDE4D (IC_50_ = 880 nM) isoforms. In pharmacokinetic studies, KVAD-88 showed good brain permeability with poor emetic effects. Furthermore, the new compound seemed to inhibit cocaine-induced hyperlocomotor activity and strikingly decreased the number of active nose-pokes and cocaine infusions in cocaine self-administering mice with differential schedules. All these data evidenced its potential for cocaine-mediated rewarding effects [169].

To reduce systemic side effects, Larsen and collaborators prepared a series of dual-soft PDE4Is for the topical treatment of atopic dermatitis. Triazolopyridine LEO39652 (IC_50_ (PDE4B) = 1.2 nM, IC_50_ (PDE4D) = 3.8 nM; Figure 10) potently inhibited TNF-α production in vitro. In preliminary in vivo pharmacokinetics studies, the derivative exhibited quick elimination in blood and the liver and long-term stability in the skin, minimizing unwanted side effects [170].

Finally, in 2021, Thirupataiah and coworkers synthesized through a PdCl_2_-catalyzed synthesis a novel class of isocoumarin-based PDE4Is. Among the isolated derivatives, **19** (Figure 10) showed (sub)micromolar potencies against PDE4B (IC_50_ = 0.54 µM) and PDE4D (IC_50_ = 1.34 µM), without cytotoxicity in MTT assays and a zebrafish embryo study. In adjuvant-induced arthritic rats, derivative **19** showed a significant reduction in paw swelling, inflammation, and pannus formation (particularly in the knee joints), as well as pro-inflammatory gene expression/mRNA levels; in addition, the compound increased body weight along with a drastic reduction in joint degenerative changes and exhibited a significant inhibitory effect on TNF-α production, highlighting its potential application in other inflammation-related diseases [171].

### 3.6. Natural Products

**Figure 11:**
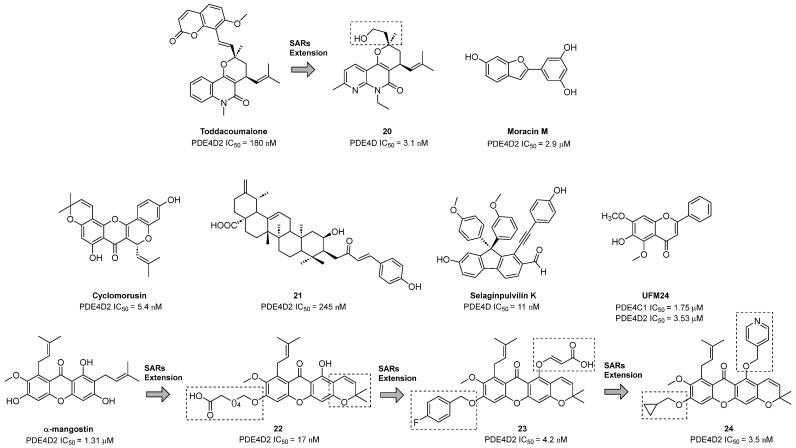
Natural PDE4DIs reported in the literature. The structural modifications of lead compounds Toddacoumalone and α-Mangostin are reported in boxes.

To date, over fifty PDE4Is have been isolated from natural sources and include terpenoid, coumarin, flavonoid, diarylfluorene, and polycyclic propylene acyl-phloroglucinol derivatives [121,149]. Toddacoumalone (Figure 11) is a mixed dimer of coumarin and quinolone scaffolds discovered in 1991 by Ishii [172]. Subsequent studies evidenced a potent inhibition of Toddacoumalone on the PDE4D2 isoform (IC_50_ = 180 nM), being more active than Rolipram [173]. To define structure–activity relationships for this compound, Song and collaborators synthesized new derivatives with high inhibitory potency and satisfactory selectivity for PDE4D. Among the prepared molecules, **20** (Figure 11) showed nanomolar IC_50_ values (3.1 nM) and remarkable therapeutic effects in an IMQ-induced psoriasis mouse model by the inhibition of TNF-α and IL-6 release. The excellent pharmacokinetic properties of the derivative support the value of this compound as a possible therapeutic agent for the treatment of psoriasis and related inflammatory diseases [174].

Extracts from *Morus alba*, a Chinese plant, were studied by Chen and coworkers for their inhibitory affinity towards PDE4. Among the considered derivatives, Moracin M (Figure 11) exhibited selective activity against PDE4D2 with an IC_50_ of 2.9 µM. Docking simulations suggested possible H-bond interactions between the compound and Gln369, Asn321, and Asp318 in the active site and further stabilization by π-π connection with Phe372 [175].

Recently, Guo and colleagues discovered Cyclomorusin (Figure 11), another derivative from *Morus alba*, which showed potent and selective inhibitory effects against the PDE4D2 isoform (IC_50_ = 5.4 nM). Molecular dynamics simulations highlighted its role as a possible pharmacophore of the prenyl moiety and the importance of the free hydroxyl groups for the activity [176].

In 2014, Cai and collaborators isolated novel triterpene derivatives from *Gaultheria yunnanensis*. Compound **21** (Figure 11) showed an IC_50_ of 245 nM against PDE4D2, resulting in the most active molecule of the series. The docking pose of **21** inside the binding pocket displayed volume changes that implied the flexibility of the PDE4 active site. The complex seemed to be stabilized by π-π interactions with Phe372 and H-bonds with Gln369 and Asn362 [177].

In 2017, Huang isolated new fluorene derivatives from the traditional Chinese medicine *Selaginella pulvinate*, which proved to comprise potent and selective PDE4DIs. In detail, Selaginpulvilin K (Figure 11), bearing a 9,9-diphenyl-1-(phenylethynyl)-9*H*-fluorene skeleton, displayed an IC_50_ of 11 nM for the PDE4D isoform; as assessed by X-ray crystallography, the compound adopted an unusual binding mode within the PDE4 binding site, with the stretched skeleton of the molecule making interactions with Q, M, and S sub-pockets and strong connections with the metal region [178].

UFM24 (Figure 11) is a 6-hydroxy-5,7-dimethoxy-flavone extracted from *Uvaria flexuosa* endowed with anti-inflammatory properties [179]. Tsai and coworkers studied the activity of the compound against PDE4 and its pharmaceutical potential in lung injuries. The derivative exhibited high selectivity for the PDE4C1 (IC_50_ = 1.75 µM) and PDE4D2 (IC_50_ = 3.53 µM) isoforms and a potent effect on human neutrophil activation. Further investigations in mice highlighted the ability of the molecule to inhibit superoxide anion and reactive oxidant generation and CD11b expression and a salutary outcome on LPS-induced acute lung injury. Furthermore, UFM24 blocked the activity of subcellular NAPDH oxidase and inhibited protein kinase B phosphorylation, further proving its potential as an anti-inflammatory agent for treating neutrophilic lung damage [180].

In 2020, using a hit-to-lead optimization approach from the natural xanthone α-Mangostin (Figure 11) from *Garcinia mangostana*, Liang identified compound **22** (Figure 11) as a potent and selective PDE4D2 inhibitor (IC_50_ = 17 nM) [181]. As evidenced by crystallographic studies, **22** adopted a unique binding pose distinct from that observed for Roflumilast and Rolipram. The compound was devoid of emetic effects and, in a vascular dementia mouse model, showed remarkable therapeutic potential at a dose of 10 mg/kg [182].

Further modifications on the xanthone scaffold led to the isolation of compound **23** (Figure 11), bearing a carboxylic moiety able to interact with the metal region in the PDE4 binding site and a benzyloxy substituent that restricted the conformational flexibility. The molecule displayed improved selectivity and potency in comparison with its analog **22** against PDE4D2 (IC_50_ = 4.2 nM) and confirmed the absence of emetic side effects on beagle dogs. The favorable pharmacokinetic properties allowed for a preliminary evaluation of the derivative in a bleomycin-induced idiopathic pulmonary fibrosis rat model, which showed comparable anti-pulmonary fibrosis activity to the reference pirfenidone [183].

Finally, the replacement of the acid group with a pyridine heterocycle and the introduction of a cyclopropylmethoxy moiety led to compound **24** (Figure 11), with higher PDE4D2 inhibitory activity (IC_50_ = 3.5 nM) and improved physico-chemical parameters. X-ray studies revealed that the molecule interacted with the M-pocket of the enzyme, showing the same key connections as the approved drug Roflumilast. When evaluated in a dextran sulfate sodium-induced inflammatory bowel disease mouse model, **24** exhibited comparable anti-inflammatory properties to the positive control dipyridamole. The improved safety and lack of emesis highlighted the pharmaceutical potential of the compound as PDE4I in inflammation-related diseases [184].

## 4. Conclusions

PDE4 enzymes hydrolyze cAMP and are involved in a number of physio-pathological processes. PDE4 inhibition increases cAMP intracellular levels, thus reducing the expression of inflammatory cytokines and enhancing regulatory cytokines; therefore, PDE4 has been evaluated as a therapeutic target in the treatment of different chronic inflammatory conditions. To date, four PDE4Is (i.e., Roflumilast, Apremilast, Crisaborole, and Ibudilast) have been approved and are currently used in therapy. A large number of PDE4Is, characterized by different chemical scaffolds, are under clinical evaluation, although their use appears to be limited by numerous side effects (e.g., diarrhea, vomiting, dyspepsia, and headache), whose onset has not been clarified yet.

**Figure 12:**
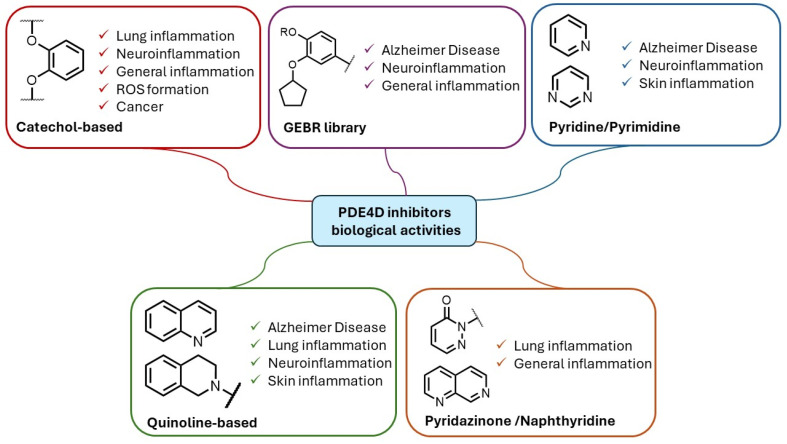
Schematic representation of the biological activities of different chemical scaffolds of reported PDE4DIs.

As reported in Figure 12, PDE4D inhibitors have been extensively investigated as potential therapeutic agents in a large variety of pathological conditions. In particular, the PDE4D isoform is highly expressed in the CNS (especially in the CA1 region of the hippocampus) and recently emerged as fundamental in cognitive, learning, and memory consolidation processes, as well as in cancer development. Selective PDE4DIs could constitute an innovative and valid therapeutic strategy for the treatment of various neurodegenerative diseases, such as Alzheimer’s, Parkinson’s, Huntington’s, and Lou Gehrig’s diseases and fragile X syndrome, but also for stroke, traumatic brain and spinal cord injury, mild cognitive impairment, and all demyelinating diseases such as multiple sclerosis. In addition, very recently, small molecules able to block the PDE4D isoform have been studied for the treatment of specific tumors, particularly in HCC and breast cancer. To date, no selective PDE4DIs have entered the market, although some of them seem to be free of the above-mentioned side effects.

In addition, the concomitant administration of PDE4DIs with other anti-inflammatory drugs represents a promising pharmacological approach to synergize the anti-inflammatory activity, reducing the required dose and minimizing the possible adverse effects.

With a very high market potential, PDE4DIs represent an attractive area of medicinal chemistry research, and the identification of novel and isoform-selective agents will enable researchers to overcome the side effects associated with the currently identified compounds.

The data here reported were collected from different databases (Scifinder, Web of Science, Scopus, Google Scholar, and Pubmed) using “phosphodiesterases” or “PDE” and “inhibitors” as keywords and considering publications (i.e., patents, reviews, research articles) published in the 1990–2023 period. Additionally, the discussion was focused on small-molecule inhibitors. The above-mentioned filters represent a limitation of this study.

## Figures and Tables

**Table 1:** Localization of the PDE isoforms in the brain regions.

PDE Family	PDE Subtype	Localization in Brain Regions *
PDE1	B	Frontal cortex, parietal cortex, temporal cortex, hippocampus, striatum
	C	Frontal cortex, parietal cortex, hippocampus
PDE2	A	Frontal cortex, parietal cortex, hippocampus, striatum
PDE4	A	Cerebellum, frontal cortex, parietal cortex, temporal cortex
	B	Cerebellum, frontal cortex, parietal cortex, hippocampus, thalamus, hypothalamus, striatum
	D	Cerebellum, frontal cortex, parietal cortex, hippocampus, thalamus, hypothalamus, nucleus accumbens
PDE8	B	Frontal cortex, parietal cortex, temporal cortex, hippocampus
PDE9	A	Cerebellum, frontal cortex, hippocampus, striatum
PDE10	A	Caudate nucleus

* Expression over 20% of maximally expressed PDE in a brain region.

**Table 2:** Structures, IUPAC names, therapeutic indications, IC_50_ values on different PDE4 isoforms, and related references of the clinically approved PDE4Is.

Compound	Structure	Condition or Disease	IC_50_	Literature Data
Roflumilast (Daxas) 3-(cyclopropylmethoxy)-*N*-(3,5-dichloropyridin-4-yl)-4-(difluoromethoxy)benzamide	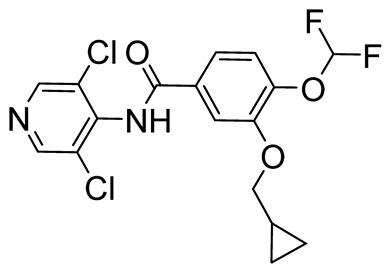	COPD	PDE4B = 0.84 nM PDE4D = 0.68 nM	[71]
Apremilast (Otezla) (*S*)-*N*-(2-(1-(3-ethoxy-4-methoxyphenyl)-2-(methylsulfonyl)ethyl)-1,3-dioxoisoindolin-4-yl)acetamide	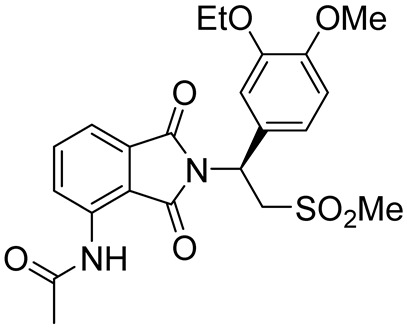	Psoriatic arthritis	PDE4 = 74 nM	[68,69]
Eucrisa (Crisaborole) 4-((1-hydroxy-1,3-dihydrobenzo[c][1,2]oxaborol-5-yl)oxy)benzonitrile	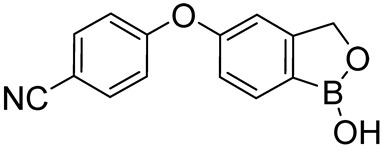	Atopic dermatitis	PDE4 = 490 nM	[68]
Ibudilast (MN-166) 1-(2-isopropylpyrazolo[1,5-a]pyridin-3-yl)-2-methylpropan-1-one	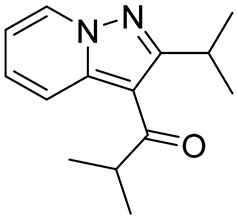	Rare childhood disease, Krabbe disease, bronchial asthma	PDE4A = 54 nM PDE4B = 65 nM	[67,68]

## Data Availability

Not applicable.

## Abbreviations

AAMI	age-associated memory impairment
AD	Alzheimer’s disease
AKAP	A-kinase-anchoring proteins
AMP	adenosine monophosphate
cAMP	cyclic adenosine monophosphate
cGMP	cyclic guanosine monophosphate
CNS	central nervous system
COPD	chronic obstructive pulmonary disease
COX	cyclooxygenase
CR	conserved region
CREB	cAMP response element-binding protein
CUS	chronic unpredictable stress
ERK	extracellular signal-regulated kinase
GMP	guanosine monophosphate
HARBS	high-affinity Rolipram binding state
HCC	hepatocellular carcinoma
IFN	interferon
IL	interleukin
iNOS	inducible nitric oxide synthase
LARBS	Low-affinity Rolipram binding state
LPS	lipopolysaccharide
MAS	McCune–Albright syndrome
MS	multiple sclerosis
PAMPA	parallel artificial membrane permeability assay
PDE	phosphodiesterase
PDE4	phosphodiesterase 4
PDE4I	phosphodiesterase 4 inhibitor
PKA	protein kinase A
RACK1	receptor for activated C kinase 1
ROS	reactive oxygen species
TNF	tumor necrosis factor
UCR	upstream-conserved region
